# Innovative Systems Biology in Baijiu Fermentation: Unveiling Omics Landscapes and Microbial Synergy

**DOI:** 10.3390/foods15050871

**Published:** 2026-03-04

**Authors:** Dandan Song, Lulu Song, Yangli Luo, Juan Chen, Chunlin Zhang, Liang Yang

**Affiliations:** 1 School of Brewing Engineering, Moutai Institute, Renhuai 564501, China; songdd0330@163.com (D.S.); songlulu@mtxy.edu.cn (L.S.); zcl818075@163.com (C.Z.); 2 Guizhou Key Laboratory of Microbial Resources Exploration in Fermentation industry, Kweichow Moutai Group, Zunyi 564501, China; ylluomoutai52@163.com; 3 School of Food Engineering, Moutai Institute, Renhuai 564501, China; chenjuan602@163.com

**Keywords:** Chinese Baijiu, multi-omics, microbial community, flavor metabolism, fermentation mechanism, stress response

## Abstract

The production of Chinese Baijiu relies on the synergistic metabolism of multi-species microbial communities in an open environment. Its intricate microbial succession and flavor formation mechanisms have long been considered complex systems that are difficult to fully deconstruct. Traditional culture-dependent techniques inherently fail to comprehensively capture the actual functional roles and dynamic regulation of “viable but non-culturable” (VBNC) microorganisms within this complex system. In recent years, the rapid advancement of multi-omics technologies has offered a novel perspective for elucidating the underlying fermentation mechanisms of Baijiu. This paper systematically reviews the recent progress in the application of metagenomics, metatranscriptomics, metaproteomics, and metabolomics in Baijiu research. Specific focus is placed on the unique contributions of these tools to resolving microbial community structural diversity, mining key functional genes and enzymes, uncovering microbial stress response mechanisms under environmental fluctuations, identifying phages and spoilage microorganisms, and tracing the metabolic pathways of flavor substances. Furthermore, the pivotal role of multi-omics integration strategies in constructing “microbe–metabolite” regulatory networks is highlighted. Finally, current challenges regarding standardization and data integration are discussed, with an outlook on leveraging omics big data to promote digital monitoring and intelligent brewing in the Baijiu industry.

## 1. Introduction

Alcoholic beverages represent some of the most ancient products of biotransformation technologies leveraged by human civilization. These traditions, such as Chinese Baijiu, have not only revolutionized dietary structures but also underpinned profound socio-political and economic implications [1,2]. Although eastern and western civilizations independently mastered fermentation techniques early in their histories, their evolutionary trajectories significantly diverged based on their disparate strategies for utilizing biocatalysts—namely, the diverse enzyme systems and microbial consortia employed to drive biochemical transformations [3,4]. Western brewing paradigms predominantly rely on malt-derived enzymes for starch hydrolysis, followed by pure-culture yeast fermentation [5]. In contrast, eastern fermentation traditions, represented by Baijiu (a traditional Chinese distilled alcoholic beverage), pioneered the use of specialized natural starters—such as “Jiuqu” (including Daqu, xiaoqu, and fuqu). These multi-functional starters contain diverse microbial communities and robust enzyme systems that facilitate simultaneous saccharification and fermentation (SSF) [6,7].

**Figure 1:**
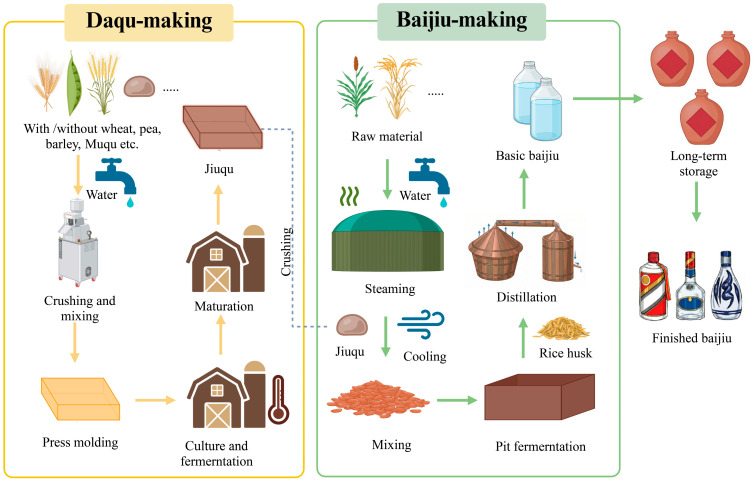
Schematic representation of the Chinese Baijiu production process. The workflow illustrates the traditional solid-state fermentation orchestrated by Jiuqu (including Daqu, Xiaoqu, and Fuqu). The key phases include: (1) Steaming: gelatinization of raw grain starch to facilitate microbial access; (2) Saccharification and Fermentation: a simultaneous process (SSF) where complex carbohydrates are converted into fermentable sugars and subsequently into ethanol and flavor compounds by diverse microbial consortia; (3) Distillation: the extraction and concentration of ethanol and volatile aromatic compounds from the fermented grain (Zaopei) using steam.

This intricate microbial consortium imparts Baijiu with its unique sensory profile and significant socio-cultural value [8,9,10]. In line with China National Standards for Baijiu production [11] (GB/T 26760-2011), Baijiu traditionally uses carbohydrate-rich grains such as sorghum, wheat, or rice. Corn, however, has been used as a substrate only in the last 3–4 centuries, following its introduction to Europe and Asia after the discovery of the Americas (Figure 1). This involves steaming, saccharification, fermentation, and distillation, all orchestrated by the starters [12]. Based on specific production techniques and sensory characteristics, Baijiu is categorized into twelve distinct flavor profiles [8,13,14].

As a quintessential element of Chinese heritage, Baijiu is not only a cultural anchor in the social fabric but also a major pillar of the national economy. According to the National Bureau of Statistics and the China Alcoholic Beverages Association, the industry’s immense vitality is reflected in its 2024 performance: the total output of Baijiu enterprises above designated size reached 4.145 million kiloliters, is approximately 4.145 million metric tons, generating a substantial sales revenue of 796.38 billion CNY (approximately 97 billion EUR). These figures underscore its status as a cornerstone of the domestic market and justify the burgeoning research interest in its biological and chemical complexity (https://www.stats.gov.cn/sj/ndsj/2024/indexch.htm, accessed on 24 February 2026).

At its core, Baijiu brewing constitutes a sophisticated, open multi-microecological system. Its distinctive sensory profile is primarily derived from the spatiotemporal dynamic succession and synergistic metabolism of functional microbial consortia, including yeasts, filamentous fungi, and bacteria [15,16,17]. However, due to the extreme heterogeneity of the solid-state fermentation matrix and the intricate nature of microbial cross-kingdom interactions, the Baijiu fermentation process has long been perceived as a highly complex system, inherently difficult to deconstruct [9,13]. While traditional culture-dependent techniques provided the initial foundation for our taxonomic understanding, they significantly struggle to capture ecological insights from the vast reservoir of microorganisms in a viable but non-culturable (VBNC). Furthermore, these classical methods are unable to systematically elucidate the causal mechanisms linking community-level functions to flavor biogenesis at the molecular level [18].

To navigate these substantial complexities, meta-omics technologies have emerged as effective frameworks designed to bridge the knowledge gap in our understanding of such intricate ecosystems. Over the past decade, the meta-omics toolkit has matured. This toolkit includes metagenomics [19,20], metatranscriptomics [21], metaproteomics [22], and metabolomics [23]. As a result, the study of complex fermentation systems has entered a new era. We now have a high-dimensional, data-intensive approach to understanding these systems [24,25,26]. These high-throughput platforms have converged the disciplines of classical microbiology and analytical chemistry, offering a systematic strategy for the holistic characterization of the Baijiu micro-ecology. By mapping the biological flow from genotype to phenotype, this integrated systems biology approach empowers researchers to investigate the previously uncharacterized microbial “black box” and metabolic landscapes of the brewing world. This allows for the accurate identification of unculturable taxa including key functional groups such as *Bacillus*, lactobacilli, *Saccharomyces*, and various non-*Saccharomyces* yeasts (e.g., *Pichia* and *Candida*), and the decoding of their in situ functional activities with high resolution [27,28,29,30].

The rapid evolution of omics technologies has catalyzed a proliferation of specialized reviews across the spectrum of fermented foods and beverages, including studies on cheese omics [31], tea omics [27], Baijiu Jiuqu [5], and beer metabolomics [32]. Despite these contributions, few studies provide a systematic integration of the entire Baijiu brewing process. Most existing literature fails to connect the biological flow from raw materials to microbial succession, and ultimately to the metabolic pathways that produce flavor. A comprehensive synthesis of these multi-omic layers covering genes, proteins, and metabolites, is essential to fully understand how biochemical transformations occur in this complex ecosystem.

Moreover, critical analyses regarding the application of omics to decipher microbial stress responses and establish predictive early-warning frameworks for spoilage are notably absent. Consequently, this review seeks to bridge this knowledge gap. Moving beyond general surveys, we focus on specific application scenarios of multi-omics within the complex Baijiu fermentation landscape. We systematically synthesize recent progress in mapping microbial diversity, mining functional gene clusters, characterizing metabolic networks, and elucidating flavor regulation mechanisms. Our objective is to provide a comprehensive conceptual framework that facilitates the transition of Baijiu production from an experience-based craft to a data-driven precision science. Furthermore, we offer critical insights into the current challenges and future prospects shaping the further development of this field.

## 2. The Microbial Landscape of Baijiu

The distinct sensory profile of Chinese Baijiu arises from a complex micro-ecological framework established through a spontaneous and open fermentation process. During production, microbial consortia are not only intentionally introduced via fermentation starters (e.g., Daqu) but are also recruited from a multitude of environmental reservoirs, including the atmosphere, soil, water, production implements, and fermentation pits [9,10,33,34,35].

The synergy between anthropogenic inoculation and natural environmental filtering ensures that the climatic conditions and biogeographic determinants of various Chinese production regions are imprinted upon specific microbial community structures. Ultimately, these communities impart highly recognizable regional sensory signatures to the spirit through their concerted metabolic activities and the transformation of raw material-derived precursors [15,35].

### 2.1. Microbial Ecology of Daqu

As a multifunctional biocatalytic carrier integrating saccharification, fermentation, and aroma production, Daqu provides a high-activity multienzyme system (comprising amylases, proteases, and esterases) and a reservoir of flavor precursors. It fundamentally dictates the sensory profile of the final spirit through its distinct micro-ecological succession and transmission [16]. Modern molecular techniques, particularly high-throughput sequencing (HTS), have demonstrated that the assembly of microbial communities in Daqu is a deterministic process rather than a stochastic one. This assembly results from the directional succession of microbiota introduced from raw materials and the environment, strictly governed by specific brewing parameters and the physical properties of the Daqu matrix [36].

Molds constitute the primary driving force for substrate bioconversion in fermentation, with the genus *Aspergillus* serving as the core functional driver. Functioning as efficient “extracellular enzyme factories,” *Aspergillus* spp. secrete potent amylase and glucoamylase systems to hydrolyze grain starch into fermentable sugars [12,37,38]. In high-temperature Daqu (HTQ), thermotolerant taxa such as *Thermoascus* spp., *Rhizomucor* spp., and *Paecilomyces* spp. emerge as the dominant mold groups responsible for maintaining saccharifying power under thermal stress [39].

While the absolute abundance of yeasts is typically lower than that of bacteria and molds, they play a decisive role in ethanol production and flavor modulation [40,41]. Although *Saccharomyces* spp. dominates the conversion of sugars into ethanol, non-*Saccharomyces* yeasts—including *Pichia* spp., *Debaryomyces* spp., and *Millerozyma farinose*, significantly enhance aromatic complexity via the Ehrlich pathway and ester biosynthesis [42].

Bacterial communities, which account for the majority of the biomass in Daqu, perform niche-specific modifications of the microenvironment through complex metabolic networks. The genus *Bacillus* constitutes the backbone microbiota across low, medium, and high-temperature Daqu types. Their secretion of diverse proteases and amylases intensifies substrate degradation, providing essential nitrogenous and carbonaceous precursors for subsequent fermentation [43,44].

Within the specialized ecological niches of High-temperature Daqu (HTQ), thermophilic groups such as *Kroppenstedtia* spp., *Oceanobacillus* spp., and *Thermoactinomyces* spp. are directionally selected; collectively, they promote amino acid metabolism and the generation of characteristic “burnt” or “sauce-flavor” compounds [45]. Conversely, in low-to-medium temperature Daqu (e.g., light-flavor types), the community structure shifts toward lactic acid bacteria (LAB), such as lactobacilli and *Weissella* spp. These acidogenic bacteria modulate the microenvironmental pH, effectively inhibiting spoilage microbes and imparting refreshing sensory characteristics to the Baijiu [46,47,48].

### 2.2. The Microbial Ecology of Pit Mud

As the distinctive fermentation interface of strong-flavor Baijiu, Pit Mud (PM) represents a highly specialized anaerobic micro-ecosystem, uniquely “domesticated” by prolonged industrial brewing cycles. Its primary biochemical hallmark lies in the biosynthesis of hexanoic acid and its subsequent esterification into ethyl hexanoate, which imparts the signature “pit aroma” to the spirit [49,50,51].

Unlike the fungal-dominated patterns observed in Daqu or fermented grains (Zaopei), the PM microbiome is predominantly prokaryotic, forming a complex syntrophic symbiotic system centered on the *Clostridia*-*Methanogen* axis. Within this consortium, members of the class *Clostridia*, including key species such as *Clostridium kluyveri*, the genus *Caproiciproducens*, and diverse *Ruminococcaceae* spp., function as the primary catalysts for hexanoic acid synthesis [52,53,54]. These anaerobes utilize ethanol and lactate as substrates to elongate short-chain fatty acids (SCFAs) into medium-chain fatty acids (MCFAs), predominantly hexanoic acid, via the reverse β-oxidation pathway.

However, this biochemical transformation is thermodynamically constrained and necessitates an obligate coupling with methanogenic partners. By acting as biological “hydrogen sinks,” methanogens—such as *Methanobacterium* spp., *Methanoculleus* spp., and *Methanosarcina* spp., maintain environmental hydrogen partial pressure at extremely low levels through interspecies hydrogen transfer (IHT) mechanisms. This thermodynamic relief effectively bypasses product inhibition, ensuring the continuous flux of hexanoic acid synthesis [55,56,57].

Beyond this functional core, PM harbors an evolving successional microbiota. While genera such as *Bacillus* spp. and *Lysinibacillus* spp. maintain a widespread but low-abundance presence, LAB, specifically lactobacilli, typically dominate the initial stages of newly constructed pit mud. As the pit matures (spanning from decades to centuries), LAB abundance gradually declines due to stringent environmental selection. Consequently, the micro-ecological assembly transitions from stochastic construction to deterministic assembly, eventually evolving into a stable climax community dominated by specialized anaerobes. This maturation process provides the ecological foundation for the traditional industry adage, “aged pits produce superior spirits” [58].

Fungal taxa are sparse in this anaerobic habitat, appearing primarily through the infiltration of Daqu or fermented grains; these include *Saccharomyces cerevisiae*, *Pichia kudriavzevii*, and *Aspergillus* spp., localized predominantly in the surface or degraded zones [53]. Furthermore, the PM microbiota exhibits pronounced spatial heterogeneity, governed by vertical gradients of moisture, ammonium nitrogen, and available phosphorus. Functional anaerobes are significantly enriched in the nutrient-dense bottom layers, leading to a sophisticated metabolic stratification phenomenon during the fermentation process [59].

### 2.3. Microbial Diversity in Baijiu Fermentation System

The implementation of modern HTS technologies has characterized the Baijiu fermentation system as an open micro-ecological reservoir defined by exceptionally high biodiversity. Current taxonomic surveys have identified more than 508 microbial species, spanning the domains of Bacteria, Fungi, and Archaea [9]. Among these, bacterial communities exhibit the highest taxonomic richness, with 335 identified species distributed across 8 phyla and 103 genera.

Within this bacterial landscape, *Bacillus* spp., *Clostridium* spp., and lactobacilli constitute the predominant taxonomic backbone. Fungal communities are primarily characterized by the genus *Aspergillus* spp., which facilitates primary saccharification, and diverse yeasts (e.g., *Saccharomyces* spp. and *Pichia* spp.) that drive the fermentation process [9]. Nevertheless, a significant portion of the ecological “dark matter” within this complex network remains poorly characterized, and its functional contribution to the brewing process has historically been underestimated.

This gap between high taxonomic richness and functional uncertainty presents a significant challenge to the precise regulation of Baijiu fermentation. While the taxonomic profiles are increasingly well-mapped, a critical knowledge gap persists regarding the actual metabolic contributions of keystone taxa and the principles of optimal community assembly [60,61,62]. Consequently, microbial metabolism during fermentation often exhibits uncontrolled stochasticity, leading to inconsistent sensory profiles across production batches [63].

Diversity analysis confined to the DNA level can no longer resolve pivotal scientific questions, such as identifying the specific microorganisms driving core metabolic flux or determining strategies to mitigate the invasion of environmental spoilage microbes. Therefore, to transcend the limitations of single-omics approaches, it is imperative to implement an integrated multi-omics strategy—encompassing metagenomics, metatranscriptomics, metaproteomics, and metabolomics.

By constructing a panoramic correlation network across the gene-transcript-protein-metabolite functional cascade, this integrated approach can resolve the latent functions of unculturable microorganisms (such as Archaea) and elucidate the coupling mechanisms between environmental drivers, microbial activity, and flavor biogenesis. Ultimately, these high-resolution insights will provide the theoretical framework required to achieve standardized control and steady-state fermentation in modern Baijiu production.

## 3. Overview of Multi-Omics Technologies Applied to Baijiu

The term “omics” signifies the holistic and systematic investigation of molecular constituents across multi-layered biological hierarchies, providing a comprehensive lens into life processes ranging from the molecular level to the macro-ecosystem scale [27]. These high-throughput approaches are designed to capture the expansive landscape of genes, transcripts, proteins, and metabolites in an unbiased, non-targeted fashion.

**Figure 2:**
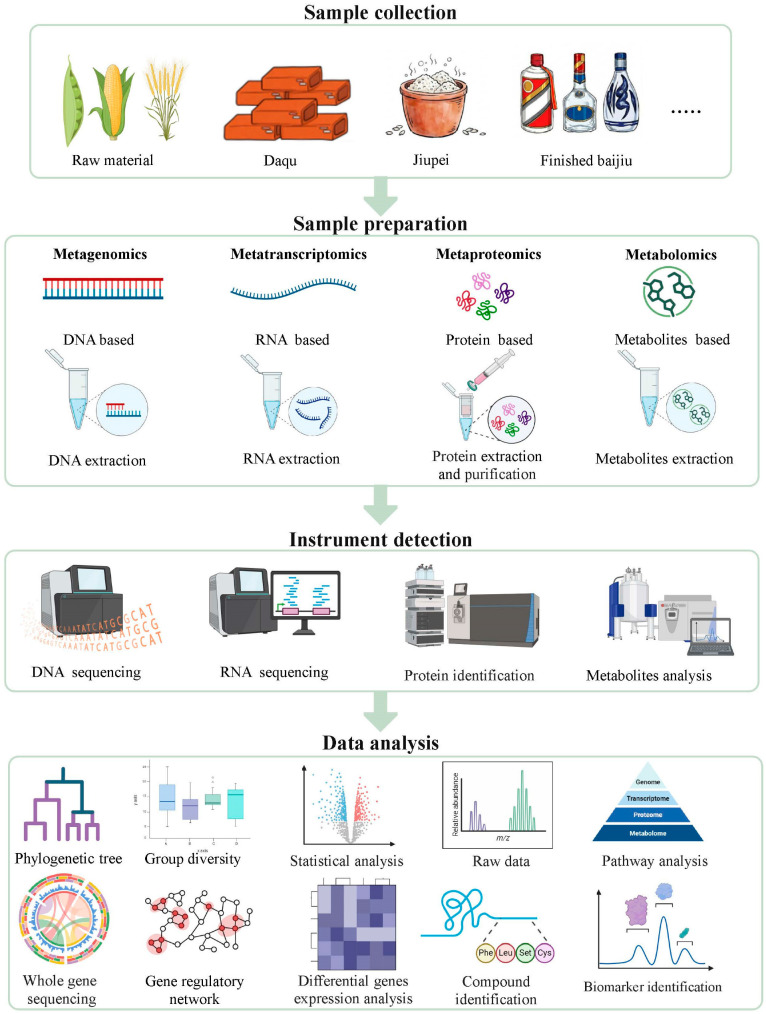
Workflows of different omics approaches, including metagenomics, metatranscriptomics, metaproteomics, and metabolomics, from sample collection to data analysis. This figure illustrates the integration of multi-omics data and the sequential steps involved in each analytical approach, highlighting the key stages such as sample preparation, sequencing, and data processing.

Consequently, they offer high-resolution molecular profiles of biological states, transcending the limitations of traditional reductive biology [26,64,65]. The maturation of robust analytical pipelines integrating optimized sampling protocols with cutting-edge hyphenated analytical platforms (e.g., next-generation sequencing (NGS) and high-resolution mass spectrometry), has paved the way for the sophisticated integration of multi-omics data (Figure 2).

In the context of traditional Chinese spirits, the synergistic application of these tools has catalyzed the emergence of a specialized discipline: “Baijiu Omics”. While this emerging systems biology framework has successfully unraveled key facets of microbial succession and metabolic flux, the inherent environmental heterogeneity and non-linear dynamic shifts in Baijiu brewing present substantial analytical challenges. Nevertheless, omics technologies remain the cornerstone for transitioning Baijiu research from purely descriptive observations toward mechanistic elucidation. This section provides a critical synthesis of the revolutionary impact of multi-omics on our understanding of Baijiu fermentation, highlighting current breakthroughs and defining the next frontier of the field (Table 1).

### 3.1. Metagenomics

Metagenomics, a field established in 1998, aims to elucidate the genomic landscapes of complex microbial consortia by directly extracting total nucleic acids from environmental matrices in a culture-independent paradigm [85,86,87]. Within the “Baijiu omics” framework, two predominant strategies are utilized: (i) Targeted Metagenomics (Amplicon Sequencing) and (ii) Shotgun Metagenomics [20,85].

Targeted metagenomics involves the directed amplification of phylogenetic anchors, such as the 16S rRNA gene for prokaryotes and the 18S rRNA or Internal Transcribed Spacer (ITS) regions for eukaryotes [85,86]. In contrast, shotgun metagenomics entails the untargeted sequencing of total community DNA, facilitating a holistic reconstruction of microbial compositions, functional gene repertoires, and metabolic blueprints [20]. The transition from single-genome analysis to large-scale metagenomic research has been catalyzed by the rapid evolution of HTS platforms, significant reductions in cost, and the exponential expansion of genomic reference repositories [87].

#### 3.1.1. Microbial Diversity and Taxonomic Resolution

Baijiu production follows an open, mixed-culture spontaneous fermentation system, where diverse microbial consortia are recruited from raw materials, Daqu, and pit mud [88]. Metagenomics has significantly advanced our understanding of microbial community succession and functional adaptation during the fermentation process, providing insights into how these microbial populations evolve and contribute to the unique characteristics of Baijiu. This high-resolution approach elucidates the spatiotemporal dynamics of microbial populations throughout the fermentation cycle.

For example, during the stacking fermentation of Baijiu, the fungal community initially dominated by *Pichia* spp., *Thermoascus* spp., *Saccharomyces* spp., and *Aspergillus* spp. gradually evolved. In parallel, the bacterial profile shifted from an initial dominance of lactobacilli and *Bacillus* spp. to a later dominance of *Acetobacter* spp., *Kroppenstedtia* spp., *Oceanobacillus* spp., and *Bacillus* spp., while *Candida* spp. became the dominant fungal genus. Microbial diversity and richness were higher at the center of the stack compared to the surface [66].

However, traditional amplicon-based methods are frequently constrained by genus-level resolution, which may lead to over-generalized ecological conclusions [20,89]. Much of the existing literature relies on simple correlations between dominant taxa and physicochemical indices [90,91], yet these associations often lack mechanistic depth and fail to establish causality. Furthermore, metataxonomic methods, such as amplicon sequencing, face inherent technical biases. While the ITS region remains a universal biomarker for fungi, non-uniform PCR amplification and intra-genomic variations in rDNA copy numbers can lead to significant abundance distortions, which are common to all amplicon-based techniques [92]. These biases are effectively bypassed by shotgun metagenomics, as total DNA is sequenced directly without the selective pressures of PCR amplification.

In comparison to amplicon sequencing, shotgun metagenomics provides unprecedented resolution for taxonomic characterization at the species and strain levels [87,93]. Beyond localized succession, it excels in deciphering the biogeographic patterns of Daqu. Evaluations based on geographic distance have confirmed a clear distance-decay relationship, where community dissimilarity expands in proportion to geographical distance. Beyond characterizing localized succession, metagenomics excels in deciphering the biogeographic patterns of Daqu. For example, evaluations based on geographic distance have confirmed a clear distance-decay relationship, where community dissimilarity expands in proportion to geographical distance [94]. Furthermore, metagenomics has been consistently employed to survey microbial communities in Daqu across various production areas [1,95,96].

Despite these transformative advantages, metagenomics faces significant interpretational challenges. A primary constraint is the inability to distinguish between the genetic potential of viable, dormant, or dead cells, potentially leading to an overestimation of the “active” diversity [29,93]. Additionally, due to the incompleteness of specialized fermentation databases, approximately 50% of environmental genes remain without functional annotation [97]. Consequently, metagenomics must be integrated with culture-dependent techniques to bridge the gap between genomic prediction and biological reality. Recent efforts in isolating target microorganisms have significantly refined species-level reference databases, enabling a more nuanced understanding of the “minority” species that often play overlooked but critical roles in flavor biogenesis [98,99,100,101].

#### 3.1.2. Functional Properties of Microbial Communities

Metagenomics has recently emerged as a critical instrument for elucidating microbial metabolic mechanisms within the open fermentation systems of Baijiu. By directly sequencing total DNA from environmental samples, this approach reveals significant correlations between taxonomic composition and functional repertoires. In contrast to amplicon sequencing, which relies on marker genes (e.g.,16S rRNA) for indirect functional inference via predictive algorithms, metagenomics facilitates the comprehensive annotation of functional genes and the in silico reconstruction of metabolic pathways through direct alignment with authoritative databases, such as KEGG and CAZy [102]. This direct methodology effectively circumvents the inherent limitations and potential biases associated with the predictive nature of amplicon-based approaches.

Guided by the “structure-determines-function” principle, researchers utilize metagenomic binning—a computational technique that clusters contigs based on intrinsic characteristics such as tetra-nucleotide frequency and GC content—to reconstruct near-complete metagenome-assembled genomes (MAGs) from environmental matrices [30,103]. This technology transcends the constraints of culture-dependency, enabling the quantification of specific microbial contributions to key metabolic pathways at the population level.

For instance, in studies of Daqu and PM, binning has substantially broadened the understanding of the functions of previously uncharacterized “dark matter” microbiota. Deep sequencing of PM samples facilitated the successful reconstruction of 703 MAGs, encompassing core phyla such as *Firmicutes* spp. (406), *Euryarchaeota* spp. (130), and *Bacteroidetes* spp. (74). This research revealed, for the first time, the potential role of *Chloroflexi* in caproic acid biosynthesis and identified *Bacteroidetes* spp. as key contributors to terpene synthesis, providing a genomic foundation for the further interrogation of PM microbial resources [67].

Another core application of metagenomics is the interrogation of whole-genome information to systematically visualize the intrinsic links between core microbiota, enzymatic activities, and metabolites. By mapping functional genes to specialized databases, researchers can resolve the functional characteristics of Daqu, the primary “biological engine” of the brewing process. In HTQ, genomic analysis systematically characterized the microbial consortia involved in starch and cellulose hydrolysis, revealing that the abundance of *Bacillus* spp. exhibited a significant positive correlation with the saccharifying power of Daqu [104]. The study further identified that specific hydrolases, such as α-amylases and glucoamylases, primarily encoded by *Bacillus* spp., *Aspergillus* spp., and *Rasamsonia* spp., serve as the core drivers of high saccharification capacity.

Microbial metabolism is integral to the flavor formation network in Daqu, which directly dictates Baijiu quality. In medium-temperature Daqu, *Lactobacillales* (mainly *Weissella* spp., lactobacilli, and *Pediococcus* spp.), *Mucorales* (mainly *Lichtheimia* spp.), and *Eurotiales* (mainly *Aspergillus* spp., *Rasamsonia* spp., and *Byssochlamys* spp.) were identified as key drivers of hydrolase production and flavor synthesis. Specifically, butanediol production was attributed to *Leuconostoc* spp., lactobacilli, and *Staphylococcus* spp., while butyric acid was derived from *Thermoactinomyces* spp. Additionally, *Byssochlamys* spp. and *Aspergillus* spp. were respectively responsible for the metabolism of guaiacol and 4-vinylguaiacol [78].

Despite these advancements, metagenomics currently faces several technical hurdles: (1) Bioinformatic Dependency: The accuracy of bioinformatic analysis depends heavily on the completeness of reference databases and the precision of annotation algorithms. (2) Database Gaps: Due to the abundance of unique, uncultured species in Baijiu brewing environments, insufficient database coverage often leads to the omission of novel functional enzymes. (3) Activity Bias: The “presence-as-activity” assumption remains a significant interpretational bias. DNA-based metagenomic sequencing reflects the genetic potential of the community but cannot distinguish between the signatures of viable, dormant, and dead microorganisms. (4) Experimental Bottlenecks: To resolve actual metabolic flux, integration with metatranscriptomics (RNA-Seq) or metaproteomics is essential. However, the inherent instability of mRNA poses stringent requirements for sample pretreatment. Effectively preventing mRNA degradation during sampling remains a primary experimental bottleneck in functional omics research today.

#### 3.1.3. Baijiu Virome

Bacteriophages and spoilage microorganisms significantly influence the stability and sensory consistency of grain-based fermentation processes [105,106]. While Baijiu research has traditionally prioritized bacterial and fungal consortia, viruses, particularly phages, frequently characterized as the “biological dark matter” of microbial ecosystems, represent a critical knowledge gap. Recently, the application of viral metagenomics has not only unveiled the immense diversity of viral communities within the Baijiu ecosystem but also elucidated their complex roles in modulating microbial succession [107,108].

Early detection is paramount for mitigating spoilage-related economic losses; however, conventional cultivation-dependent methods are often hindered by laborious protocols and low throughput [24,109]. Metagenomic sequencing offers a culture-independent, high-throughput alternative for characterizing phage sequences directly from complex matrices [24,106]. As the biological “engine” of fermentation, Daqu serves as a primary reservoir for viruses [9]. Studies utilizing viral DNA extraction and taxonomic profiling have revealed that in HTQ, *Parvoviridae* is the predominant viral family, whereas *Genomoviridae* prevails in medium- and low-temperature variants [110]. Furthermore, significant abundance fluctuations in phages belonging to *Siphoviridae*, *Podoviridae*, *Herelleviridae*, and *Myoviridae* have been observed across different *Daqu* types [110]. During the critical five-day stacking phase, which enriches functional microbes, the virome is primarily composed of *Siphoviridae* and *Inoviridae*. Upon transitioning to pit fermentation, the viromic landscape shifts toward a dominance of tailed phages [111,112].

Despite these advances, characterizing the Baijiu virome remains a significant bioinformatic challenge. First, the ubiquitous presence of host genomic DNA interferes with the bioinformatic extraction of viral signals, frequently leading to false-positive identifications due to high sequence homology between prophages and bacterial genomes [106,108,113]. Second, the scarcity of comprehensive viral reference repositories hinders the functional annotation of assembled viral contigs. Resolving these hurdles requires advanced algorithms capable of identifying novel viral sequences within complex datasets and elucidating their ecological roles within the fermentation microbiome [24,105,114,115,116,117]. Two major hurdles remain: identifying viral genomic sequences within complex metagenomic datasets and elucidating the ecological roles of these assembled viral genomes within the fermentation ecosystem.

Currently, Baijiu viromics is in its infancy. Future research should transition from simple sequence mining toward establishing specialized Baijiu viral resource banks. Validating host-infection dynamics will be essential to close the loop from “genomic prediction” to “functional verification”.

#### 3.1.4. Spoilage Microorganisms and Off-Odors

Beyond viruses, metagenomics is an indispensable tool for deciphering the origins of amine-like off-flavors produced during *Daqu* production. By integrating molecular sensory science with metagenomic mining, trimethylamine (TMA) has been identified as a primary contributor to amine-like odors in HTQ, with *Bacillus* spp., *Kroppenstedtia* spp., and *Thermoascus* spp. recognized as the key functional drivers [118].

Another detrimental sensory defect is the “pickled vegetable” smell, linked to volatile sulfur compounds such as dimethyl trisulfide (DMTS) and 2-furfuryl-mercaptan [119]. While metagenomics identifies the genetic “potential” for spoilage, confirming active metabolic flux requires a multi-omics integrative approach. Studies tracking *Yan-cai* odorants have successfully coupled metagenomic predictions with culture-dependent validation to confirm the odor-producing capacity of specific isolates [120]. Metagenomics has further revealed that spoilage microbes exhibit highly active metabolism in pathways such as sulfur relay systems and cysteine/methionine metabolism, providing a molecular basis for early-warning detection [119,121,122].

For spoilage control, the integration of metagenomics with metatranscriptomics is recommended to assess the actual in situ activity of pathogens, thereby enhancing the precision of quality control in the modern Baijiu industry. Additionally, leveraging these advanced multi-omics approaches will enable more targeted interventions, optimize fermentation processes, and help predict and mitigate spoilage issues before they impact the product quality. As the field of metagenomics continues to evolve, the application of these tools will be essential in transforming Baijiu production into a more controlled, efficient, and scientifical-driven process, ensuring consistent flavor profiles and improving product safety.

### 3.2. Metatranscriptomics

While DNA-based metagenomics effectively maps taxonomic profiles and metabolic potential, a fundamental limitation remains: its inability to differentiate between transcriptionally active microorganisms and those that are structurally intact but metabolically quiescent or represent extracellular “necromass” [27,29]. Metatranscriptomics circumvents this constraint by directly capturing environmental RNA signatures, thereby identifying functionally active community members and the specific metabolic pathways expressed under defined spatiotemporal conditions [21,79]. This high-resolution functional mapping provides critical biological insights inaccessible via DNA-level analysis, particularly in discerning transient metabolic states and real-time microbial responses to the fluctuating fermentation environment [21,123].

The standard metatranscriptomic workflow comprises five pivotal stages: environmental RNA extraction, mRNA enrichment (e.g., rRNA depletion), high-throughput sequencing, bioinformatic data processing, and metabolic visualization [114,124].

Within the context of Baijiu fermentation—a process characterized by extreme acidity and high polysaccharide interference—metatranscriptomics serves as a robust tool for identifying differentially expressed genes (DEGs). It offers unprecedented insights into the in situ functional activities of the active microbiota. In recent years, this approach has been instrumental in elucidating metabolic flux, uncovering microbial stress response mechanisms (such as thermo- or acid-tolerance), and investigating the regulatory impacts of bacteriophages on the transcriptional profiles of key fermentative strains. By synchronizing the “potential” identified by metagenomics with the “activity” captured by metatranscriptomics, researchers can construct more accurate models of the biochemical transformations driving Baijiu quality.

#### 3.2.1. Metabolic Pathways

Research into Baijiu fermentation has historically faced challenges in accurately capturing the in situ functional activities of microbial communities. This is due to the inherent limitations of cultivation-dependent methods and the static nature of DNA-based metagenomics, which can only characterize genomic potential. In contrast, metatranscriptomics provides a more dynamic approach, enabling the resolution of active microbial functions at the transcriptional level. By sequencing environmental RNA and aligning it with functional databases, metatranscriptomics offers direct insights into the biosynthesis of flavor precursors and the actual metabolic flux occurring during brewing.

During Daqu fermentation, the thermal trajectory of the pile acts as a primary environmental filter. As temperatures rise to 50 °C and 62 °C, thermolabile taxa typically undergo metabolic dormancy or cell death, whereas thermotolerant groups exhibit significant upregulation of key enzymes involved in glycolysis, starch degradation, and pyruvate metabolism. Furthermore, the substantial enrichment of transcripts associated with the tricitric acid cycle (TCA cycle) at 62 °C facilitates the accumulation of diverse organic acid derivatives essential for aroma development [70]. This thermal selection partially explains why the aroma profiles of medium-temperature Daqu (MTQ) and HTQ are significantly richer than those of low-temperature Daqu (LTQ) [125]. Comparative studies underscore that while elevated heat favors the expression of flavor-related metabolic pathways, it often necessitates a trade-off with the community’s overall saccharifying and ethanologenic capacities [126]. Notably, metatranscriptomic evidence has highlighted the pivotal role of lactobacilli in driving the biosynthesis of esters and their essential fatty acid precursors [71].

Beyond static snapshots, metatranscriptomics offers unique advantages in deconstructing the spatiotemporal dynamics of the fermentation ecosystem. A “stage-specific metabolic reprogramming” model has been proposed for sauce-flavor Baijiu. In this framework, transcriptional activity is predominantly channeled toward ethanol synthesis during the initial phase, followed by a dramatic shift in metabolic flux toward the accumulation of lactic and acetic acids in the subsequent stage [127]. Similarly, in MTQ fermentation, a “microbial relay race” is observed, genera such as *Lachnoclostridium* spp., *Staphylococcus* spp., and *Pichia* spp. drive the early energy-releasing processes, while thermotolerant filamentous fungi (e.g., *Rhizopus* spp. and *Lichtheimia* spp.) dominate the late-stage transcriptional landscape. These fungi function as both primary saccharifying agents and producers of aromatic compounds, bridging the gap between enzyme activity and the final aroma profile of mature Daqu [39].

The precise reconstruction of metabolic networks necessitates high-fidelity enzyme annotation (e.g., via EC numbers). By linking enzymes participating in the same metabolic modules, researchers can visualize the complex interplay between microbial populations and chemical outputs [128]. However, “transcript-to-metabolite” inference remains partially predictive. To achieve a holistic understanding of the Baijiu micro-ecology, it is essential to perform orthogonal validation using metaproteomics or metabolomics.

Crucially, integrated metatranscriptomic analysis has unveiled the niche differentiation of transcriptionally active host communities. Fungal populations exhibit peak activity in LTQ, whereas bacterial communities dominate the transcriptional landscape in MTQ and HTQ. This synergistic validation between viral composition and host metabolic activity provides a novel perspective for understanding the biological regulation of the fermentation micro-ecology. The deep coupling of transcriptomic landscapes with detailed metabolic phenotypes remains the gold standard for systematically elucidating the mechanisms governing traditional Baijiu fermentation.

#### 3.2.2. Baijiu Virome

In complex micro-ecosystems such as Baijiu fermentation, bacteriophages execute host infection and survival strategies through the orchestrated expression of key infection-related transcripts and virulence-associated genes. Compared to the static genetic blueprints provided by genomics, metatranscriptomics precisely reveals the dynamic expression patterns of both phage and host genes during the infection cycle, serving as an indispensable tool for deciphering virus–host interaction mechanisms [129,130].

The application of metatranscriptomics in studying spoilage microorganisms is currently nascent, yet it offers transformative potential for precision quality control. Research presently focuses on temperature-mediated niche partitioning of viruses across various Daqu types. Recent multi-omics investigations have dissected the ecological interactions within the Daqu microbiome, demonstrating that fermentation temperature significantly reshapes viral community structures. Specifically, viromic profiling revealed that *Parvoviridae* is the predominant viral family in HTQ, whereas *Genomoviridae* prevails in medium-temperature and low-temperature variants (MTQ/LTQ) [110]. Furthermore, significant abundance fluctuations have been observed in phages belonging to *Siphoviridae*, *Podoviridae*, *Herelleviridae*, and *Myoviridae* across different thermal zones.

However, existing studies are largely confined to general viromic surveys. Research on the specific spoilage microorganisms responsible for acidification, off-flavors, or yield reduction in Baijiu remains scarce, with a notable lack of investigation into the transcriptional states, survival strategies, and stress-response mechanisms of these detrimental strains. Future metatranscriptomic research should prioritize specific “spoilage bacteria–phage” interaction pairs. By coupling transcriptomic signatures with phenotypic observations through orthogonal validation, researchers can construct a comprehensive logical framework spanning from molecular triggers to macroscopic outcomes. This approach will facilitate the development of predictive early-warning models and mechanistic analyses of the microbial spoilage process, ultimately ensuring the steady-state stability of modern Baijiu production.

#### 3.2.3. Stress Response and Interactions

Baijiu fermentation represents a dynamic spatiotemporal succession governed by fluctuating environmental stressors, including organic acid accumulation, thermal shifts, high osmotic pressure, and nutrient deprivation [2,8,131]. The metabolic plasticity of the constituent microbiota is primarily regulated by the differential expression of genes induced by these selective pressures. By comparing the transcriptional landscapes of fermenting strains under steady-state versus stressed conditions, metatranscriptomics enables the identification of genome-wide molecular signatures underlying the stress response. This facilitates the elucidation of survival and adaptation strategies employed by microbes within extreme solid-state fermentation (SSF) environments [114].

During the Baijiu brewing process, fermentation temperatures frequently exceed 50 °C due to exothermic microbial metabolism or environmental constraints. Under such thermal stress, *Saccharomyces cerevisiae*, a primary producer of flavor compounds gradually loses viability [132]. In contrast, *Pichia kudriavzevii* mitigates high-temperature damage by upregulating energy metabolism, specifically the oxidative phosphorylation pathway, to increase ATP production. As the core of energy metabolism, the tricarboxylic acid (TCA) cycle is significantly activated in these thermotolerant taxa, supplying increased NADH and FADH_2_ for oxidative phosphorylation [133].

Furthermore, the accumulation of organic acids exerts significant pressure on microbial growth. When *P. kudriavzevii* and *S. cerevisiae* are co-cultured, *P. kudriavzevii* upregulates genes associated with lactate degradation and H^+^ efflux. This creates a favorable “metabolic window” for *S. cerevisiae*, enhancing lactic acid consumption efficiency far beyond that achieved in monocultures and increasing overall tolerance to lactate stress [134]. These findings confirm at the transcriptional level that the “catabolism-transport regulation” pathway is a critical route for yeasts to maintain intracellular pH homeostasis.

Exogenous nutrient supplementation has also been shown to activate acid tolerance genes. For instance, serine supplementation significantly improved glucose transport and cellular structural integrity (>30%) in *Zygosaccharomyces bailii*. It also upregulated the expression of genes associated with the recovery of mitochondrial membrane potential, correspondingly increasing intracellular ATP levels (+296.6%) and ethanol yield (+226.6%) [72]. This research successfully established a correlation model of “gene transcription-metabolic homeostasis-fermentation performance”.

Maintaining micro-ecological stability is vital during fermentation, as excessive mold growth can lead to the “caking” or “clumping” of fermented grains (Zaopei). The antifungal effect of phenylethanol, a metabolite produced by *Pichia* spp. via the Ehrlich pathway, on *Monascus purpureus* helps mitigate this phenomenon. Metatranscriptomic analysis has identified potential antifungal mechanisms, including the inhibition of protein synthesis and the induction of DNA damage [73,135].

In summary, metatranscriptomics provides a molecular window into the profound stress-response mechanisms of microorganisms, facilitating the discovery of stress-resistant phenotypes and transcriptional signatures in high-performance strains. However, the ultimate goal of scientific exploration lies in industrial application. Future research should focus on translating these omics-driven discoveries into practical process strategies, such as precision nutrient supplementation or the design of synthetic microbial consortia. Such interventions can effectively alleviate the impact of environmental stress on functional strains, thereby maximizing the economic returns of Baijiu production at an industrial scale.

### 3.3. Metaproteomics

While metagenomics and metatranscriptomics provide comprehensive insights into microbial composition and metabolic potential, enzymes constitute the definitive catalytic machinery of the fermentation process. Because neither DNA- based nor RNA-based approaches can directly characterize actual enzymatic activity, an in-depth analysis at the protein level is essential [29]. Although metatranscriptomics reveals gene expression levels and cellular dynamics, the functional regulation of these activities is ultimately governed by the expressed proteome. Consequently, taxonomic profiling alone cannot unequivocally identify the specific taxa responsible for the metabolism of Baijiu raw materials. Furthermore, mRNA levels frequently exhibit non-linear correlations with protein abundance due to pervasive post-translational regulation and varying half-lives; thus, discrepancies between transcriptomic predictions and metaproteomic realities are common in Baijiu research [22,136].

Proteomics, a concept introduced by Wilkins and Williams in 1994, refers to the large-scale study of the entire protein complement expressed by an organism, tissue, or cell under specific physiological conditions [29]. By mapping protein expression landscapes, this technology resolves metabolic pathways and molecular interactions while providing insights into community-level functional proteostasis [137]. In food science, metaproteomics offers critical perspectives on niche partitioning and enzymological characteristics, serving as an indispensable tool for deciphering fermentation dynamics and the biochemical principles of flavor formation [138].

Methodologically, metaproteomics relies on sophisticated analytical platforms. The maturation of high-resolution separation technologies—such as liquid chromatography (LC) and isotope-coded affinity tags (ICAT)—coupled with breakthroughs in high-resolution mass spectrometry (HRMS) and database-dependent search algorithms, now allows for deep-proteome profiling of complex food matrices [29,139].

A standard “bottom-up” metaproteomic workflow typically encompasses five pivotal stages: (1) Protein Extraction and Purification: Tailored to overcome the high polysaccharide and phenolic interference in Baijiu fermented grains. (2) Enzymatic Digestion: Typically utilizing trypsin to generate a complex peptide pool. (3) MS-based Peptide Analysis: Utilizing platforms like Orbitrap or TOF for high-sensitivity detection. (4) Protein Identification and Quantification: Employing rigorous FDR control to ensure taxonomic and functional accuracy. (5) Computational Systems Biology: Integrating differential expression analysis, functional annotation (GO/COG), and Protein–Protein Interaction (PPI) network modeling [22,140,141].

#### 3.3.1. Protein Expression Profiles and Functional Landscapes

To address central scientific challenges such as the taxonomic-functional decoupling inherent in genomic predictions and the ambiguous biological origins of key functional enzymes, metaproteomics provides definitive evidence by systematically profiling the functional proteostasis of microbial communities [142]. Compared to metagenomics and metatranscriptomics, this technology circumvents functional inference biases based solely on sequence homology. It enables the discovery of novel functional enzymes and the precise source-tracking of their biological origins, effectively bridging the gap between genetic potential and active enzymatic machinery [29].

PM harbors a vast array of anaerobic microorganisms essential for the biogenesis of hallmark flavor compounds [143]. Metaproteomic analysis comparing PM across chronological gradients (30/300 years) revealed 59 highly expressed proteins associated with flavor formation in centuries-old pits. Specifically, methanogens and key anaerobes such as *Clostridium* spp. and *Methanobacterium* spp. were found to be highly correlated with the synthesis of organic acid precursors (butyric, hexanoic, and acetic acids). These findings suggest that protracted maturation periods promote the accumulation of stable enzymatic landscapes, thereby enhancing spirit quality [144]. Due to the architecture of the fermentation pit, protein expression exhibits significant spatial heterogeneity. For instance, proteins involved in ADP and purine nucleoside diphosphate metabolism show peak variance across different PM strata, likely driven by gradients in available phosphorus (AP). AP concentrations significantly modulate the proteomic profiles of *Paenibacillus* spp., *Kroppenstedtia* spp., and *Nocibacillus* spp., illustrating the sensitivity of the proteome to environmental micro-niches [74].

As the primary “biological engine” and a multi-functional bio-catalytic carrier, Daqu contributes an integrated system of microbes, raw materials, enzymes, and flavor precursors to the brewing process [9,145]. Despite its significant protein content, research has historically prioritized taxonomic composition or metabolomic profiling, leaving the metaproteomic landscape of Daqu largely under-explored [146,147].

Recent methodological breakthroughs have utilized orthogonal experimental designs to optimize protein extraction protocols specifically for the complex Daqu matrix. While early studies focused on the fluctuations of five foundational enzymes: glucoamylase, cellulase, pectinase, protease, and esterase, across the core and surface of Daqu, comprehensive metaproteomic characterization during the full fermentation cycle remains a frontier [29,148]. Initial assessments of stored Daqu successfully identified 17 protein spots representing 16 distinct proteins from bacteria, yeasts, and filamentous fungi, though their specific metabolic roles and precise taxonomic assignments warrant deeper investigation [149].

In contrast, a greater number of enzymatic proteins were detected in low-temperature Daqu, Metaproteomic analysis of low temperature Daqu revealed a functional landscape comprising 422 distinct enzyme proteins, with their expression levels primarily distributed across oxidoreductases (121) and transferases (108). High-intensity expression was specifically noted in alcohol dehydrogenase among oxidoreductases, as well as starch synthase and β-amylase within the transferase and hydrolase groups, respectively. These elevated expression profiles align with the metabolic requirements for ethanol biosynthesis and starch hydrolysis, which are fundamental to the fermentation efficiency of this specific Daqu variety [77].

During fermentation, protein expression in Daqu displays distinct spatiotemporal dynamics. Enzyme and total protein levels are initially enriched in the upper layers, but as the process matures, the middle layer emerges as the functional epicenter with the highest expression levels [30]. Crucial redox systems, including superoxide dismutase (SOD), glyceraldehyde-3-phosphate dehydrogenase (GAPDH), and aldehyde dehydrogenase (ALDH), maintain peak expression throughout the cycle, ensuring cellular homeostasis under fermentation stress. These patterns indicate that the middle-layer microbial community possesses both a robust structural assembly and superior functional proteostasis [30].

The proteomic profile of HTQ is also modulated by storage and seasonality. Post-storage, the bacterial community dominates the functional landscape, with total and enzymatic protein levels measured at 1.46 and 1.48 times higher than their fungal counterparts, respectively [150]. While protein abundance typically peaks in summer, enzyme expression varies by class. Generally, the abundance of hydrolases (EC 3), lyases (EC 4), and isomerases (EC 5), aligns with the seasonal cycle, whereas most enzymes—except for ligases (EC 6)—exhibit significantly attenuated expression in winter Daqu. While current research has advanced our mechanistic understanding, metaproteomics still faces hurdles regarding database coverage due to uncharacterized “dark matter” organisms. Future investigations should prioritize integrating multi-omics validation with de novo sequencing to enhance protein identification resolution. Such approaches will ultimately bridge the gap between genomic potential and real-world enzymatic functionality, facilitating precise control of the Baijiu fermentation process.

#### 3.3.2. Stress Response and Microbial Adaptations

Throughout the dynamic succession of Baijiu fermentation, microbial communities are persistently subjected to complex environmental filters, including nutrient depletion, extreme temperature fluctuations, and pronounced spatial heterogeneity. Metaproteomics reveals how these microorganisms maintain cellular proteostasis by modulating translation efficiency and remodeling protein expression profiles, providing direct molecular evidence for adaptive evolution within the fermentation ecosystem [151]. Specifically, key enzymes involved in ADP and purine nucleoside diphosphate metabolism exhibit strong spatial specificity within dominant taxa such as *Paenibacillus* spp., *Kroppenstedtia* spp., and *Nocibacillus* spp. This metabolic preference serves as a direct physiological response to localized phosphorus limitation. Fine-tuning metabolic flux without necessarily altering protein abundance has emerged as a sophisticated strategy for microorganisms to cope with such nutrient scarcity [74].

Beyond spatial constraints, temperature fluctuation is the predominant environmental factor shaping the functional landscape of the community. Microorganisms primarily employ a dual strategy of taxonomic succession and metabolic reinforcement to combat thermal stress. Research has confirmed the niche-filtering effect of temperature on functional microbiota, saccharification in medium-temperature environments relies on the thermolabile *Lichtheimia ramosa*, whereas high-temperature stress selectively recruits the thermotolerant *Desmospora* spp. to execute chitinase secretion. This species replacement ensures functional continuity and resilience throughout the process [152].

Seasonality also profoundly impacts enzymatic abundance. During the high-temperature summer season, *Eurotiales* spp. and other fungal groups in “yellow Daqu” significantly upregulate the expression of cellulase, α-amylase, and glucoamylase compared to low-temperature seasons. This heat-induced enzymatic upregulation empowers the microbiota with enhanced substrate degradation capabilities, meeting the accelerated metabolic demands of the summer fermentation cycle [75]. Furthermore, temperature-driven climatic fluctuations govern the deterministic assembly of microbial communities through homogeneous selection. High temperatures in summer enhance the abundance of *Bacillus velezensis* and *Bacillus licheniformis*, activating their maltogenic α-amylase and cyclomaltodextrinase secretion systems. This process promotes starch metabolism centered on maltose, facilitates acetate phosphorylation and acetyl-CoA generation, and consequently reduces sugar content and acidity. Conversely, low temperatures in winter favor the dominance of *Pediococcus acidilactici*, indicating that it plays a role in acid accumulation through the expression of lactate dehydrogenase [153].

Metaproteomics has preliminarily decoded the “genotype-proteotype-phenotype” associations of Baijiu microbiota responding to environmental stress. However, current research remains largely descriptive, focusing on functional enrichment patterns. Future investigations must establish multi-dimensional integration frameworks, encompassing metatranscriptomics and metabolomics, to systematically elucidate the deep-seated metabolic regulatory networks and signaling pathways of microorganisms within these extreme fermentation environments.

#### 3.3.3. Identification of Key Functional Microorganisms

Metaproteomics facilitates a precise transition from a simple “taxonomic inventory” to “core functional mapping” by capturing the dynamic expression of proteins at the translational level. This approach provides direct evidence for the actual metabolic roles of microorganisms, effectively bridging the functional gap between genetic potential and real-world enzymatic activity.

In investigations aimed at identifying “key saccharifying microbiota” for Daqu quality enhancement, researchers initially screened eight bacterial and seven fungal genera as predominant taxa using high-throughput amplicon sequencing. However, subsequent metaproteomic validation revealed that only five of these candidates significantly contributed to the functional protein profile. Lactobacilli, *Pichia* spp., and *Rhizopus* spp. were identified as the primary sources of glycosidases and glycosyltransferases. α-Amylase and glucoamylase, originating from *Aspergillus* spp., *Rhizomucor* spp., and *Rhizopus* spp., were characterized as the two essential enzyme families driving starch hydrolysis and correlating with ethanol yield [37,38]. Interestingly, comparative analysis indicated that a decrease in *Daqu* “porosity” (manifested as increased bulk density) significantly inhibited the metabolic activity of these saccharifying microbes. This suggests that porosity and bulk density serve as critical physical drivers for the functional expression of the saccharifying proteome.

During the fermentation process, temperature gradients drive the differentiation of *Daqu* into three distinct types: White, Yellow, and Black. Integrated metaproteomic and metabolomic analyses identified five-membered heterocyclic amino acids as the key metabolic markers driving this micro-ecological divergence. Correspondingly, *Neurospora crassa*, *Aspergillus nidulans*, *Bacillus subtilis*, and *Oceanobacillus iheyensis* were identified as “chameleon microorganisms”. These versatile taxa modulate the metabolic flux of heterocyclic amino acids, ultimately mediating the niche partitioning of high-temperature Daqu (HTQ) micro-ecology [154,155].

In the study of flavor components, regarding ester production, the hallmark flavor compounds of strong-flavor Baijiu, metaproteomics confirmed that *Aspergillus* spp., *Bacillus* spp., *Leuconostoc* spp., and *Pediococcus* spp. exhibit significantly high expression of ester-synthesis enzymes at both the transcriptional and translational levels. A synergistic consortium centered on *Aspergillus* spp. dominates the ester metabolic network in medium-high temperature Daqu (MHT-Daqu) [76].

Similarly, an integrated analysis utilizing metagenomics and metaproteomics unveiled the acetaldehyde metabolic architecture in Jiang-flavor Baijiu fermentation, characterized by two PDC-mediated biosynthetic pathways and three NADH-ADH-mediated conversion pathways. A critical temporal asynchrony was observed: the activation of aldehyde-producing PDC preceded that of aldehyde-consuming NADH-ADH during stacking, resulting in substantial acetaldehyde accumulation that was not effectively metabolized until NADH-ADH was upregulated upon entry into the pit. Functional profiling pinpointed *Schizosaccharomyces* pombe and *Saccharomyces cerevisiae* as the primary metabolic drivers. Furthermore, the study clarified specific regulatory interactions, showing that *Pichia kudriavzevii* enhances acetaldehyde synthesis via interspecies synergy, whereas Aceti lactobacilli *jinshanensis* suppresses the reduction pathway through environmental acidification [156].

Metaproteomics effectively compensates for the inherent limitations of functional prediction associated with amplicon sequencing. It confirms that within the complex microbial consortia of Baijiu brewing, only a fraction of core taxa act as the actual drivers of critical saccharification and esterification processes. Furthermore, this technology provides deep insights into how physical factors (e.g., porosity) and characteristic metabolites (e.g., heterocyclic amino acids) regulate microbial metabolic activities, elucidating the intrinsic mechanisms of Daqu micro-ecological differentiation.

While functional enrichment analysis is currently the mainstream approach to studying environmental impacts on microbial physiology, research on physical structure-driven functional expression remains in its infancy. Future studies should prioritize the integration of metaproteomics with metatranscriptomics and metabolomics to construct a “panoramic correlation network” (Species–Gene–Protein–Metabolite). Such a framework will further resolve the stress adaptation strategies of microorganisms in complex brewing environments and enable the precise regulation of flavor metabolic phenotypes.

### 3.4. Metabolomics

Since the establishment of its conceptual framework in 1998, metabolomics has evolved into a central pillar of systems biology. As the proximal representation of the functional phenotype bridging genotypes and environmental interactions, this technology enables the comprehensive qualitative and quantitative profiling of low-molecular-weight metabolites (< 1500 Da) within complex biological systems. It provides an unparalleled perspective for decoding the intricate biochemical regulatory networks inherent in the Baijiu fermentation ecosystem [23,25,157].

As research focus has shifted, the application of metabolomics has expanded from the physiological characterization of single strains to the elucidation of the collective metabolic phenotypes of microbial communities [141]. Metabolomic strategies are broadly categorized into targeted and non-targeted approaches. Targeted metabolomics: prioritizes high quantitative specificity, sensitivity, and reproducibility for a predefined set of metabolites (e.g., organic acids or specific esters). Non-targeted metabolomics aims to maximize the coverage of the metabolic landscape, making it the preferred tool for discovering potential bioactive compounds and unknown flavor precursors [158,159,160].

Given the extreme complexity of the Baijiu fermentation matrix, single analytical platforms are often insufficient. Consequently, the integration of modern instrumentation such as gas chromatography-mass spectrometry (GC-MS) for volatile compounds and liquid chromatography-mass spectrometry (LC-MS) for polar or non-volatile metabolites—has significantly expanded detection dimensions and depth. These hyphenated techniques are now mainstream tools for resolving the chemical divergence of Baijiu across the production chain, from raw materials to the finished Baijiu [161,162].

A standard metabolomic workflow encompasses four core stages: sample collection, metabolite extraction (including quenching), data acquisition, and chemometric analysis. Following raw data acquisition, sophisticated bioinformatic pipelines resolve complex metabolic networks through preprocessing algorithms (e.g., peak picking and alignment), metabolite annotation (using libraries such as NIST or HMDB), and multivariate statistical modeling, including Principal Component Analysis (PCA) and Partial Least Squares-Discriminant Analysis (PLS-DA) [163,164,165]. Applying metabolomics to Baijiu brewing facilitates the characterization of biochemical trajectories underlying microbial succession and elucidates the mechanistic logic of microbe-microbe interactions driving flavor generation.

#### 3.4.1. Dynamic Changes and Characterization of Metabolites

In the open SSF system of Chinese Baijiu, metabolomics serves as a core strategy for deciphering the functional phenotypes of microbial communities rather than merely being a tool for quantification [166]. Microorganisms transform substrates through intricate metabolic networks into a diverse array of compounds, including alcohols, esters, acids, and aldehydes. The fluctuations of these metabolites characterize the biochemical trajectories of the microbiota. Compared to conventional volatile profiling, metabolomics simultaneously captures a comprehensive landscape, encompassing non-volatile organic acids, polyphenols, and carbohydrates, thereby constructing high-fidelity metabolic fingerprints for fermentation monitoring [154].

The interplay between environmental variables and metabolic responses is a critical research focal point. Seasonal precipitation and temperature significantly drive metabolic phenotype differentiation in strong-flavor *Daqu*. High temperatures and humidity in summer promote the metabolic activity of *Bacillus* spp., leading to the synthesis of pyrazine-based roasted aroma compounds. Conversely, lower winter temperatures drive the enrichment of ester flavor compounds [167]. Specific environmental conditions in spring Daqu have also been linked to increased guaiacol accumulation [168].

During Daqu preparation, metabolites exhibit dynamic shifts categorized into three distinct physiological phases: (1) early stage (days 0–4): primary nutrient accumulation. This phase is dominated by the accumulation of non-volatile organic compounds (non-VOCs). High saccharifying power drives a surge in reducing sugars (e.g., glucose) and acetic acid. (2) middle stage (days 7–15): flavor compound transformation. This is a critical window for the formation of volatile organic compounds (VOCs), during which ethanol, ethyl acetate, and phenylacetaldehyde accumulate significantly. (3) Late stage and maturation: metabolic homeostasis. Reducing sugars decline due to microbial consumption, and metabolic focus shifts toward functional substances such as branched-chain aldehydes and tetramethylpyrazine. Continuous Maillard reactions and enzymatic catalysis further enhance the abundance of polar metabolites like amino acids and esters, promoting flavor balance [79,169]. Similar spatiotemporal variations in both volatile and non-volatile metabolites are observed during the stacking and pit fermentation processes of Baijiu [170,171,172,173].

Geographical origin results in distinct chemical signatures, making the identification of biomarkers via non-targeted metabolomics essential for authenticity and quality control. Strong-flavor Baijiu is primarily categorized into two regions: the Sichuan Basin (SCB) and the Yangtze-Huaihe River Basin (YHRB). SCB samples typically contain higher concentrations of pyrazines (e.g., trimethylpyrazine) and furans (e.g., 2-acetyl-5-methylfuran), imparting intense roasted and sauce-flavor (*Jiang-xiang*) notes. This is attributed to higher Daqu incubation temperatures (50–60 °C) and fermentation temperatures (28–35 °C), which accelerate Maillard reactions. In contrast, YHRB samples exhibit higher levels of 3-methylbutyric acid, various esters (e.g., isobutyl acetate), and alcohols (e.g., 1-butanol). Lower fermentation temperatures (25–32 °C) in this region help retain short-chain esters, providing pronounced fruity notes. A total of 22 biomarkers, including isobutyl acetate, dimethyl disulfide, and dimethyl trisulfide, serve as key indicators to distinguish these two regional styles [174].

Flavoromics has further resolved the synergistic logic underlying Baijiu blending. Utilizing technologies such as GC × GC-TOFMS, researchers identified 577 volatile compounds and 47 key odorants (OAV > 1), clarifying the complementary effects between the “ester-acid backbone” of base liquor and the “aging notes” of flavoring liquor. Base Liquor (Dazongjiu, 80%): constitutes the structural foundation, contributing pit aroma, alcoholic sensation, and roasted notes. Flavoring Liquor (Tiaoweijiu, 0.1%): highly correlated with the finished product and critical for imparting aged, fruity, and refreshing characteristics. Substitute Liquor (Daijiu, 15%): Enriches the profile with grainy, floral, and herbal notes. This transition from a “static component list” to a “dynamic association network” provides precise metabolic phenotypic information for process optimization [175].

Despite the analytical power of metabolomics, the chemical complexity of Baijiu presents significant hurdles. A primary bottleneck is the incomplete coverage of fermentation-specific secondary metabolites in public databases, which hinders accurate annotation. Furthermore, the scarcity of commercial standards for many characteristic metabolites limits absolute quantification. Future research must integrate multi-dimensional chromatography with the construction of localized proprietary databases to overcome these identification barriers in complex matrices [164].

#### 3.4.2. Metabolic Pathway Elucidation and Functional Decoding

In interpreting high-dimensional metabolic data, a discrete inventory of differential metabolites (DMs) often fails to capture the underlying logic of complex biological processes. Consequently, metabolomics serves as a “systemic decoder” in Baijiu research. By constructing high-dimensional microbe–metabolite co-occurrence networks, this approach effectively decouples multi-species metabolic interactions within complex fermentation matrices. Furthermore, pathway enrichment analysis enables the identification of key biochemical modules, precisely elucidating the mechanisms of flavor biogenesis and sensory phenotype evolution at the molecular level [176,177]. This paradigm shift, from “single biomarker screening” to “system-level pathway analysis”—allows researchers to establish mechanistic causality between functional molecules and macroscopic sensory phenotypes [178,179].

Pathway analysis, originally developed for transcriptomics, has evolved into a cornerstone of metabolomic interpretation. This method relies on structured databases, most notably KEGG (Kyoto Encyclopedia of Genes and Genomes), to provide a comprehensive map of biological transformations. Such analysis is particularly valuable for tracking the biotransformation of specific precursors in Baijiu systems [180]. For instance, KEGG enrichment revealed that furfurylthiol, a critical sulfur-containing odorant is a product of the cysteine metabolism pathway, synthesized from cysteine via thioester intermediates [80]. While reconstructing a complete metabolic network necessitates the integration of gene expression data and orthogonal validation [181,182].

At the mechanistic level, enrichment analysis of specific pathways can pinpoint the biochemical routes responsible for unique visual or sensory characteristics. Non-targeted metabolomics has been used to deconstruct melanin formation in HTQ. A significantly active tyrosine metabolism pathway directly correlates with enzymatic browning and melanin accumulation, providing a molecular basis for the unique appearance of “Black Daqu” [183]. In addition, metabolomics is also analyzed for flavor phenotypes. Enrichment analysis identified phenylalanine metabolism, pyruvate metabolism, and glycolysis as core drivers of “douchi-aroma” (fermented soybean-like scent) in HTQ. Within the phenylalanine pathway, the final product—phenylacetic acid—imparts this characteristic aroma [176].

Despite the power of non-targeted metabolomics, inferring in situ functions from a single-omics perspective remains limited. Baijiu fermentation is a complex ecosystem governed by multi-strain co-cultures and intricate gene regulation. Metabolite abundance alone cannot distinguish between de novo microbial synthesis and non-enzymatic biotransformation, nor can it define actual genotype expression levels. Therefore, to construct high-fidelity metabolic regulatory networks, future research must move beyond single-omics observations. By integrating targeted metabolomics, metatranscriptomics, and metaproteomics, researchers can achieve a profound leap from metabolic phenotypic observation to “gene-to-metabolite” regulatory elucidation.

#### 3.4.3. Quality and Safety Inspection

Food safety represents a critical concern that directly impacts consumer health and institutional trust. Governance failures in this area can result in significant reputational damage and economic losses for distilleries [184,185]. Within food safety research, metabolomics is driving a paradigm shift from the “static monitoring” of individual contaminants toward a “mechanistic deconstruction” of risk-factor migration and quality evolution within complex matrices.

By integrating metabolomics with chemometrics, researchers have systematically mapped the spatiotemporal migration profiles of microbial contaminants throughout the brewing chain. Non-targeted metabolic profiling has identified harmful metabolites such as ethyl carbamate (EC), which remain critical areas of investigation for food safety [186,187].

Beyond exogenous risk control, food-omics strategies based on high-resolution mass spectrometry (HRMS) act as “molecular detectives” for authenticity identification and quality grading. By monitoring differential metabolites, including organic acids and esters, researchers can effectively distinguish the metabolic fingerprints of naturally aged Baijiu from those subjected to γ-ray irradiation-accelerated aging. This approach also enables real-time tracking of the degradation kinetics of harmful byproducts like carcinogenic plasticizers under specific interventions [82].

Research on light-flavor Baijiu further indicates that product grade transitions correspond directly to the optimization of metabolic constituents. As quality grades increase, the ester-to-alcohol ratio becomes more balanced, imparting enhanced floral and fruity notes. Notably, ethyl acetate has been identified as a dual-marker for both grade classification and geographical origin. Geographical origin profoundly shapes these metabolic fingerprints: northern Baijiu tends to be enriched with acidic compounds, leading to pronounced sourness, whereas southern samples exhibit a more vigorous floral profile due to a higher proportion of esters and ketones [184].

The integration of metabolomics with machine learning (ML) is transitioning quality evaluation into a digital and intelligent paradigm. Utilizing flavoromics strategies assisted by models such as Multilayer Perceptron (MLP) and XGBoost, researchers have achieved up to 97% accuracy in classifying the quality grades of different fermentation rounds in sauce-flavor Baijiu.

By incorporating SHAP (Shapley additive explanations) interpretability models, these studies have further defined the critical concentration thresholds governing grade transitions. For example, diethyl succinate (2.30 mg/L) has been identified as a benchmark for acidic aroma quality, while tetramethylpyrazine plays a dominant role in optimizing sauce-like (20.66 mg/L) and burnt (15.36 mg/L) flavor characteristics. This depth of research—transitioning from “abundance detection” to “evolutionary mechanisms”—not only enhances the precision of food safety analysis but also provides a systemic theoretical foundation for industry standards through the construction of “microbiota–metabolism–safety–grade” dynamic association networks [188,189].

### 3.5. Multi-Omics Strategies and Data Integration in Baijiu Research

The advent of high-throughput technologies has expanded our understanding of the bioprocesses underlying Baijiu fermentation. By integrating metagenomics, metatranscriptomics, metaproteomics, and metabolomics, multi-omics strategies provide a holistic view of gene expression, protein translation, and metabolic flux, which are crucial for elucidating the complex relationships that govern flavor biogenesis [138]. Unlike single-omics studies, multi-omics approaches overcome the limitations of isolated data by constructing systemic correlations across multiple biological layers, offering deeper insights into the mechanisms of spontaneous fermentation [24,31].

To translate high-dimensional omics data into actionable insights, various integration strategies have been developed. Traditional statistical models, such as Redundancy Analysis (RDA) and Canonical Correspondence Analysis (CCA), are effective for identifying associations between microbial features and chemical outputs; however, these methods distinguish correlations rather than direct causality.

To circumvent these limitations, cross-tier interaction network modeling has been implemented. For instance, high-resolution networks have resolved the links between flavor components, functional genes, and microbial populations in sauce-flavor Baijiu [190].

Early results indicate that ML algorithms such as Support Vector Machines (SVM) and Random Forests (RF) have been successful in predicting key flavor components and microbial interactions, showing promise for improving fermentation control [191,192,193,194]. Nevertheless, the inherent noise and incompleteness of molecular interaction data remain significant constraints. In response, ML algorithms are increasingly leveraged to extract non-linear patterns and reveal hidden regulatory architectures. While ML application in fermentation is nascent, the success of SVM, Random Forests (RF), and Causal Bayesian Networks in related disciplines provides a robust blueprint for Baijiu research [193,195,196,197]. The primary challenge lies in the industrial validation of these ML-driven insights to ensure their efficacy in real-world production environments. Recent studies suggest that these techniques can significantly improve the prediction of fermentation outcomes and quality control parameters, but large-scale industrial trials are needed for validation.

Multi-omics integration has provided crucial insights into the biological mechanisms underlying Baijiu fermentation. For instance, the integration of metabolomics with metagenomics and metatranscriptomics has enabled the identification of keystone taxa responsible for the degradation and formation of phthalate esters (PAEs) in the fermentation process [198,199,200]. By constructing specialized search databases using metagenomic sequences, metaproteomics significantly enhanced the identification of proteins from previously unculturable taxa, which are critical to understanding the microbial diversity of Baijiu fermentation [22,30]. These findings underscore the effectiveness of multi-omics in elucidating the complex microbial interactions and metabolic pathways involved in flavor formation and spoilage control.

At the phenotypic level, the concurrent application of transcriptomics and proteomics clarifies translational efficiency and the consistency of gene expression. In the ester metabolic network of Daqu, for example, *Aspergillus* exhibits a high degree of correlation and consistency between its transcriptional activity and protein-level functional output [76]. In summary, multi-omics integration serves as the essential bridge connecting the micro-ecological structure of Baijiu fermentation with its flavor functionality. Although challenges remain in the standardized integration of heterogeneous data, the optimization of bioinformatic algorithms and system-level modeling will further deepen our understanding of these dynamic regulatory networks, providing a solid scientific foundation for intelligent and standardized Baijiu production.

## 4. Current Challenges and Future Perspectives

Omics strategies have been widely adopted as pivotal frameworks for elucidating the mechanistic complexities, the so-called “black box” of Baijiu fermentation, providing profound systemic and biological insights. Collectively, the convergence of multi-layered omics approaches has empowered the scientific community to capture a multi-dimensional array of high-resolution data across diverse fermentation stages.

**Figure 3:**
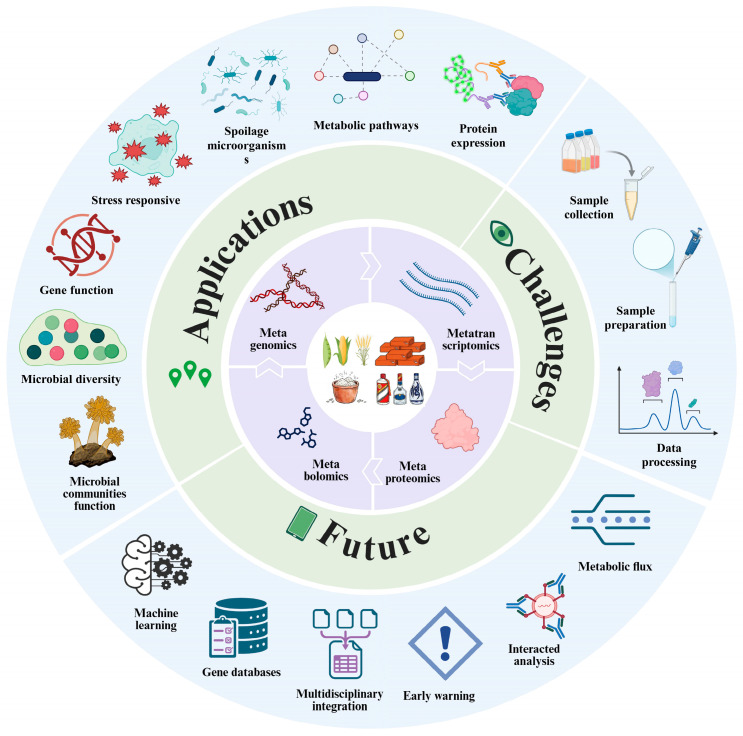
Applications, challenges, and future perspectives of omics technologies in Baijiu research.

However, despite these significant advancements, several major challenges remain to be addressed before the analytical maturity of “Baijiu Omics” can be fully realized. Key bottlenecks include the standardization of heterogeneous data, the limited coverage of specialized fermentation databases, and the inherent difficulty in establishing causal links within such a complex, spontaneous ecosystem. Nevertheless, the future outlook for this field is highly optimistic. We are currently witnessing a pivotal transition from purely descriptive observations toward more data-driven, predictive, and intelligent methodologies (Figure 3). The integration of artificial intelligence and systems biology is poised to revolutionize the industry, paving the way for a new era of precision brewing.

### 4.1. Current Challenges in Baijiu Multi-Omics

Although multi-omics technologies have made transformative strides in resolving biological complexities, their effective integration within the specialized SSF matrices of Baijiu remains hindered by multifaceted bottlenecks. These challenges span the entire analytical pipeline, ranging from the complex physicochemical properties of upstream samples to the significant hurdles of downstream data standardization.

#### 4.1.1. Matrix Interference and Extraction Efficiency

A primary obstacle is the development of extraction strategies tailored to the exceptionally complex lignocellulosic matrix of Baijiu. Unlike animal-derived fermented products, such as yogurt or cheese, Baijiu and its fermented grains (Zaopei) constitute a plant-based system characterized by high concentrations of polysaccharides, polyphenols, and recalcitrant secondary metabolites. These matrix constituents severely compromise the extraction efficiency and purity of high-quality nucleic acids and proteins [9,138].

In genomics and transcriptomics, polysaccharides act as potent inhibitors of Taq polymerase and restrict endonucleases, while oxidized polyphenols can undergo covalent cross-linking with nucleic acids. Compounded by the inherent hydrolytic instability of RNA, particularly the rapid turnover of mRNA, obtaining high-integrity samples without co-extracting inhibitors remains exceptionally difficult [65,87]. While alternative high-throughput extraction methods have been proposed to avoid hazardous reagents, they often suffer from low purity, poor recovery, and inconsistent downstream amplification. Consequently, the optimization of “matrix-compatible” extraction protocols remains a critical pursuit [85].

#### 4.1.2. Proteomic and Metabolomic Obstacles

For metaproteomics and metabolomics, an ideal extraction framework must ensure comprehensive coverage while minimizing metabolic degradation and protein denaturation. This objective necessitates the precise optimization of cell lysis kinetics and buffer-to-sample ratios [23,29].

In metaproteomic profiling, plant-derived secondary metabolites significantly interfere with protein isolation. Phenolic compounds can form irreversible complexes with proteins, establish strong hydrogen bonds with peptide backbones, or react with thiol (–SH) and amino (–NH) groups. Therefore, the strategic elimination of these interfering phenolics is a prerequisite for effective protein recovery from Daqu or Zaopei [88,201,202,203].

In metabolomics, the challenge manifests as a trade-off between extraction breadth and selectivity. Because the Baijiu metabolome encompasses chemically divergent substances, ranging from highly volatile esters to polar carbohydrates and amino acids, no single solvent system can achieve comprehensive capture without inducing chemical artifacts or degradation. Establishing standardized metabolic quenching and dual-phase extraction schemes is essential for the future of systemic fermentation interpretation [164,204,205].

#### 4.1.3. Bioinformatic Hurdles and Lack of Standardization

Beyond physical barriers, limitations in data processing and the absence of methodological standardization constitute a “secondary technological wall”. During bioinformatic annotation, the scarcity of comprehensive, high-confidence reference databases, specifically for the “dark matter” of uncharacterized Baijiu microbiota, poses a significant risk of false-positive identifications and the accumulation of unannotated “orphan” genes. Furthermore, the seamless fusion of heterogeneous multi-omics data, characterized by differing dimensions and vast dynamic ranges, remains a daunting computational hurdle.

Most critically, the comparability of research findings is compromised by non-standardized experimental designs. Regional variations in Baijiu production, fluctuations in process parameters, and raw material heterogeneity lead to high baseline noise in omics results. The field currently lacks unified sampling frameworks and consistent data acquisition protocols. This lack of rigor not only weakens the reliability of individual studies but also hinders large-scale meta-analysis and cross-validation between research teams.

### 4.2. Future Directions and Perspectives

To address the inherent challenges of matrix interference and data fragmentation within Baijiu fermentation, future research must transcend the mere accumulation of single-omics datasets. The field is poised for a paradigm shift toward multi-technique cross-disciplinary fusion and advanced knowledge-driven data mining.

#### 4.2.1. Enhancing Spatiotemporal Resolution and Multi-Modal Characterization

A critical priority is the integration of omics analysis with advanced physical and biochemical imaging to overcome the current limitations in spatiotemporal resolution. Baijiu fermentation is a dynamic process characterized by extreme micro-environmental heterogeneity; traditional “homogenized” sampling often masks critical micro-scale metabolic discrepancies.

The introduction of mass spectrometry imaging (MSI), high-resolution electron microscopy, and advanced spectroscopy will provide essential molecular topography regarding spatial metabolite distribution and microscopic structural shifts. Furthermore, the application of Metabolic Flux Analysis (MFA) will facilitate the dynamic quantification of intracellular carbon and nitrogen fluxes. This “map-spectrum-activity” integration strategy not only validates omics-based inferences but also elucidates the microscopic mechanisms of microbial metabolism in situ, providing a visual mechanistic explanation for complex macroscopic fermentation phenomena.

#### 4.2.2. AI-Driven Data Fusion and Predictive Modeling

As high-dimensional datasets proliferate, the capacity to extract biological value will depend heavily on the integration of explainable artificial intelligence (AI) and deep learning. To address current data “silos”, future work should focus on developing algorithms for the multimodal fusion of heterogeneous multi-omics datasets.

By constructing AI-driven predictive models, researchers can achieve a seamless interface between genotypes and phenotypes, establishing robust early warning systems for pathogens and spoilage microorganisms throughout the supply chain. This big-data-driven risk assessment mechanism can monitor the successional trajectories of micro-ecological communities to predict their impact on final product sensory profiles and safety, thereby enabling proactive intervention and precision quality control.

#### 4.2.3. Regional Ecological Decoding and Database Construction

Baijiu research must return to its essence as a “regional ecological fermentation” process. Given the inadequacies of general-purpose microbial databases, it is imperative to construct region-specific genomic and metabolomic repositories that encompass functional strains tailored to specific production areas and raw materials.

Future research should focus on deconstructing the internal coupling mechanisms of the quaternary system: “raw Materials–microorganisms–environment–metabolites”. Investigating how different substrates (e.g., specific sorghum cultivars) and environmental variables shape microbial niche construction will determine the characteristic *terroir* of the spirit. This will provide a “theoretical compass” for designing personalized fermentation processes based on specific environmental inputs.

#### 4.2.4. Industrial Valorization and Biosafety Frameworks

The ultimate goal of multi-omics research is the rational design and industrial application of Synthetic Microbial Consortia. However, this progress must be advanced within a rigorous biosafety and ethical framework. While metabolic engineering and fortified starter cultures hold immense potential for enhancing the microbial synthesis of bioactive compounds, such as specific terpenes or pyrazines, the ecological risks of introducing non-native or modified strains into open fermentation environments must be strictly managed.

Biosafety assessments must be integrated throughout the entire lifecycle, from in silico R&D to large-scale industrial implementation. In conclusion, the future of Baijiu research will be a systemic undertaking that integrates multi-dimensional characterization, intelligent computation, and ecological regulation, facilitating the leap from traditional spontaneous brewing to a digital, high-precision modern bio-manufacturing technology.

## 5. Conclusions

In summary, the application of multi-omics technologies has catalyzed a transformative paradigm shift in Baijiu research, facilitating a decisive transition from traditional “black box” empirical exploration toward data-driven mechanistic elucidation. As detailed in this review, metagenomics has established the foundational genomic blueprint of microbial diversity, identifying core functional drivers and potential spoilage risks. Furthermore, the integration of metatranscriptomics and metaproteomics has dynamically decoded the functional expression of these communities, revealing how environmental stressors and inter-species interactions regulate metabolic activities in real-time. Finally, metabolomics serves as the proximal representation of the biochemical phenotype, mapping the intricate networks of flavor compounds and secondary metabolites formed throughout the fermentation cycle.

Crucially, the leap from single-omics observations to integrated multi-omics strategies represents the most significant milestone in the field. By correlating genotypic potential with phenotypic endpoints, researchers can now construct precise regulatory networks that directly link specific microbial taxa to key flavor biogenesis. Although challenges regarding heterogeneous data standardization and the functional validation of “dark matter” microorganisms persist, the developmental trajectory of the field is clearly oriented toward systemic integration.

As these high-throughput technologies continue to mature, coupled with the profound synergy of artificial intelligence (AI) and systems biology, they will undeniably lay a robust scientific foundation for the standardization of Baijiu quality, the prediction of fermentation outcomes, and the ultimate realization of intelligent, high-precision brewing for the traditional Chinese Baijiu industry.

## Figures and Tables

**Table 1:** Application of multi-omics in Baijiu fermentation.

Omic Approach	Sample	Summary of the Results	Reference
Metagenomics	Daqu	This study utilized metagenomics to elucidate the microbial and metabolic differences among various Daqu types from seven provinces. Distinct core functional microbiota were found to drive specific metabolic pathways, including the generation of esters, alcohols, and acids—thereby dictating the functional differentiation responsible for flavor formation.	[45]
Metagenomics	Jiupei	Resolved spatio-temporal heterogeneity during stacking fermentation of Sauce-flavor Baijiu. Revealed that the pile core exhibited higher diversity with round-dependent succession (e.g., lactobacilli to *Acetobacter*), while the pile surface enriched thermotolerant microbes (e.g., *Kroppenstedtia*). Moisture and pH were identified as key drivers for community assembly in the core and surface, respectively.	[66]
Metagenomics	Pit mud	Reconstructed 703 Metagenome-Assembled Genomes (MAGs) via deep sequencing, identifying core taxa including *Firmicutes* (406), *Euryarchaeota* (130), and *Bacteroidetes* (74).	[67]
Metagenomics	High-temperature Daqu	Identified a core functional group comprising 7 bacterial and 4 fungal genera (including *Kroppenstedtia*, *Thermoactinomyces*, *Aspergillus*, etc.). *Bacterial* genera were found to have the most significant impact on Daqu flavor.	[68]
Metagenomics	Low-temperature Daqu	Detected 1286 genera with a bacteria-to-fungi ratio > 4:1 (*Bacillus* dominant). Revealed functional complementarity among three Daqu types: Houhuo Daqu dominates flavor generation, while Qingcha and Hongxin Daqu specialize in macromolecule degradation to support fermentation.	[69]
Metatranscriptomics	Medium-temperature Daqu	lactobacilli, *Staphylococcus*, and *Pichia* were metabolically active in the early stage. Thermotolerant filamentous fungi became transcriptionally active in the high-temperature/late stage, serving as key saccharifying agents and producers of aromatic compounds.	[39]
Metatranscriptomics	Jiuqu	Identified fungi as the most active community members. CAZymes and glycolysis/starch metabolism enzymes were highly expressed at 50 °C and 62 °C. Upregulation of TCA cycle enzymes at 62 °C was critical for flavor derivative formation	[70]
Metatranscriptomics	Jiupei	Quantified absolute abundance of LAB using internal standards. Confirmed LAB as active functional microbes significantly correlated with eight flavor compounds, actively transcribing genes for flavor biosynthesis.	[71]
Metatranscriptomics	Jiupei	Demonstrated that serine upregulates glucose transport and cell structure genes in lactate-stressed *Zygosaccharomyces bailii*. This transcriptional reprogramming restored membrane integrity (>30%) and mitochondrial potential, significantly boosting ATP (+296.6%) and ethanol yield (+226.6%).	[72]
Metatranscriptomics	Jiupei	Identified the Ehrlich pathway as the primary route for 2-phenylethanol synthesis by *Pichia*. Revealed potential antifungal mechanisms involving inhibition of protein synthesis and induction of DNA damage.	[73]
Metaproteomics	Pit mud	Key enzymes for ADP and purine metabolism in dominant taxa (*Paenibacillus*, *Kroppenstedtia*, *Nocibacillus*) showed strong spatial specificity. This metabolic bias was identified as a direct response to local phosphorus limitation.	[74]
Metaproteomics	Daqu	Seasonal temperature differences significantly affected enzyme abundance. Compared to winter, *Eurotiales* and other fungi in summer Daqu significantly upregulated cellulase, α-amylase, and glucoamylase expression.	[75]
Metaproteomics	Jiuqu	Identified functional saccharifying enzymes in situ. lactobacilli, *Pichia*, and *Rhizopus* were core contributors of glycosidases. α-amylase and glucoamylase (from *Aspergillus*, *Rhizomucor*, and *Rhizopus*) were confirmed as key enzymes for starch hydrolysis and ethanol production.	[38]
Metaproteomics	Medium-high-temperature Daqu	*Aspergillus*, *Bacillus*, *Leuconostoc*, and *Pediococcus* highly expressed ester synthesis enzymes at both transcriptional and translational levels. An *Aspergillus* centered synergistic community dominated the ester synthesis metabolic network.	[76]
Metaproteomics	Daqu	Identified 422 enzymes, saccharifying enzymes showed the highest activity among hydrolases. Correlation analysis indicated a positive relationship between *Erwinia* and saccharification power.	[77]
Metabolomics	High-temperature Daqu	Identified key volatile compounds defining Daqu flavor during incubation, including 3-methylbutanol, 1-hexanol, 1-octen-3-ol, phenylethyl alcohol, ethyl hexanoate, hexanal, and benzaldehyde.	[78]
Metabolomics	High-temperature Daqu	Fresh Daqu showed significant differences in amylase and protease activities between the inner and outer layers. After storage, the metabolic profiles of the inner and outer layers converged and became similar.	[79]
Metabolomics	Jiupei	KEGG enrichment analysis revealed that furfuryl thiol, a key sulfur compound, is synthesized from cysteine via thioester intermediates, identifying it as a critical product of the cysteine metabolic pathway.	[80]
Metabolomics	Raw materials, Finished baijiu	Screened 12 core pollutants via non-targeted metabolomics. Elucidated the distribution of plasticizers across the raw material-fermentation-distillation chain and revealed the dynamic distribution of DBP and DEHP during distillation.	[81]
Metabolomics	Base baijiu	Monitored 29 differential metabolites during aging. Distinguished metabolic fingerprints between natural aging and γ-irradiation, and tracked the degradation of carcinogenic plasticizers under specific interventions.	[82]
Metabolomics	Medium-temperature Daqu	Identified 139 compounds across six stages. Esters formed in early-mid stages, while pyrazines appeared in late stages. Key aroma compounds were identified, including guaiacol, 4-ethyl-2-methoxy phenol, and various pyrazines.	[83]
Metabolomics	Low-temperature Daqu	Identified acetate, betaine, choline, 1,7-dimethylxanthine, proline, erythritol, lactate, arabinitol, and syringate as chemical biomarkers for low-temperature Daqu.	[84]

## Data Availability

The original contributions presented in the study are included in the article, further inquiries can be directed to the corresponding author.

## Abbreviations

The following abbreviations are used in this manuscript:

Abbreviation	Full name
VBNC	Viable but non-culturable
HTS	High-throughput sequencing
HTQ	High-temperature Daqu
LAB	Lactic acid bacteria
PM	Pit Mud
SCFAs	Short-chain fatty acids
MCFAs	Medium-chain fatty acids
IHT	Interspecies hydrogen transfer
ITS	Internal Transcribed Spacer
MAGs	Metagenome-assembled genomes
TMA	Trimethylamine
DMTS	Dimethyl trisulfide
MTQ	Medium-temperature Daqu
LTQ	Low-temperature Daqu
SSF	Solid-state fermentation
LC	Liquid chromatography
ICAT	Isotope-coded affinity tags
HRMS	High-resolution mass spectrometry
PPI	Protein–Protein Interaction
AP	Available phosphorus
SOD	superoxide dismutase
GAPDH	glyceraldehyde-3-phosphate dehydrogenase
ALDH	aldehyde dehydrogenase
GC-MS	gas chromatography-mass spectrometry
LC-MS	liquid chromatography-mass spectrometry
PCA	Principal Component Analysis
PLS-DA	Partial Least Squares-Discriminant Analysis
VOCs	Volatile organic compounds
SCB	Sichuan Basin
YHRB	Yangtze-Huaihe River Basin
DMs	Differential metabolites
PAEs	Phthalate esters
EC	Ethyl carbamate
ML	Machine learning
MLP	Multilayer Perceptron
RDA	Redundancy Analysis
CCA	Canonical Correspondence Analysis
SVM	Support Vector Machines
RF	Random Forests
MSI	Mass Spectrometry Imaging
MFA	Metabolic Flux Analysis
AI	Artificial intelligence
